# Electrical Osmosis for the Treatment of Living Dentine

**Published:** 1896-02

**Authors:** Henry W. Gillett

**Affiliations:** Newport, R. I.


### International Dental Journal.

Vol. XVII. February, 1896. No. 2.

### Original Communications.[1]

### ELECTRICAL OSMOSIS FOR THE TREATMENT OF LIVING DENTINE.[2]

BY HENRY W. GILLETT, D.M.D., NEWPORT, R. I.

Mr. President and Gentlemen,—Electrical osmosis, electrical diffusion, anodal diffusion, and cataphoresis are terms used by different authorities to express nearly the same phenomena.

The first three terms will be recognized as entirely synonymous, and need no defining to make them intelligible.

Electrical osmosis, the one I have chosen for my title this evening, is probably the one having the widest acceptation among electricians.

Cataphoresis is a medical term which has come into use among electrotherapeutists, and is recognized by few electrical experts outside of those interested in medicine.

The definition of the term given by Dr. William J. Morton[3] seems to cover the ground fully. It is this:

"The movements of fluids, together with the substances they

[1] The editor and publishers are not responsible for the views of authors of papers published in this department, nor for any claim to novelty, or otherwise, that may be made by them. No papers will be received for this department that have appeared in any other journal published in the country.

[2] Read before The New York Institute of Stomatology, November 26, 1895.

[3] Cataphoresis and solution of $H_2O_2$ for bleaching teeth, etc., Dental Cosmos, June, 1895. Note also the terms electric diffusion and electric medicamental diffusion, proposed by Dr. W. J. Morton.

hold in solution, from the positive pole of electrodes conveying a continuous current in tissue towards the negative pole."

When we consider this definition in connection with the human tooth, which generations of our profession have been striving to penetrate with some drug which should modify its sensitiveness, or with applications which should modify morbid conditions of the tooth-pulp, it becomes at once important, and, indeed, imperative that we make use of this principle, if we find it possible to do so. It is not necessary for me to mention before this audience the many advantages to accrue, for both patient and operator, from any feasible and harmless method of subduing the sensitiveness of the dentine of the human tooth.

It is equally unnecessary for me to enumerate the many different means that have been tried to attain this end. It has not always happened that the means which have been tried and found helpful have proved harmless. The ill results from the use of arsenic and the strong mineral acids are examples of this fact. I have been amazed, within a year or two, to find a set of these so-called obtundents endorsed by members of our profession of recognized standing, which are of a most pernicious character. Upon applying to them simple tests within the reach of every intelligent operator, they reveal the fact that their efficiency is due to the most corrosive mineral acids.

Of course, every progressive operator has certain applications which he uses with success in some percentage of cases, but I think it is the universal experience to find that, in many of the cases where we need help most, none of these applications are of much assistance.

For several years I have been taking a keen interest in a method of applying drugs to sensitive dentine, which seemed to promise a more universal usefulness than anything which we have before had at our command.

I approached the matter with great hesitation because of my lack of knowledge of electrical principles. Finally, however, I found the key which simplified the problem for me. It has taken much labor to reduce the matter to a point where it is practicable. Since March I have been able to command, for daily use, a process for the control of the sensitiveness of dentine, which seems to be practically universal in its application, and from which I can conceive no possibility of ill results. As I go on, I find others have considered the same possibilities.

The earliest efforts along this particular line that I have been able to hear of are those of Dr. D. F. McGraw,[1] now of California. I understood Dr. McGraw to have discarded the idea because of defects arising from crudeness of apparatus, and it is just that point which marks the dividing line between success and failure.

The apparatus needs to be specially adapted to our needs. The living dentine and tooth-pulp are many times more sensitive to the electrical current than most other tissues. It is consequently necessary that the current should be under the operator's complete control, so as to allow him to modify it by adding or withdrawing very small amounts.

Dr. A. C. Westlake, of this city, approached this subject later, but has given us no practical results, so far as I have learned.

Dr. John S. Marshall, of Chicago, has worked along a kindred line, but up to last August had not attempted to control sensitiveness of dentine.

As I look over my own papers on the subject, presented at Asbury Park, last August (one of which will appear in the *Dental Cosmos* later), I find that I have progressed, and that some of my statements need to be modified. Many points need to be restated, and others to be amplified, while still other points, about which I could then only guess, have now come within the realm of positive knowledge.

With your permission, however, I wish to go back to the earliest recorded experiments demonstrating the facts of electrical osmosis in living tissue.

The records of these experiments have been very satisfactorily collected, and added to by Dr. Frederick Peterson, and I wish to quote freely from his article in the recently published "International System of Electro-Therapeutics."[2] He concludes his definition of cataphoresis with the statement that it seems to be a purely physical process. These are his words:

"Physically and not physiologically speaking, it is almost certain that electrical endosmosis is a mechanical and not an electrolytic effect, for two reasons: first, the action will show itself in any single solution whenever a porous partition, such as plaster of Paris, stoneware, etc., is inserted in the path of the current; even when no electrolysis is taking place,—that is, no decomposition; and, secondly, because the phenomenon may be stated as follows: When-

---

[1] Dental Review for 1888.

[2] An International System of Electro-Therapeutics, by Horatio R. Bigelow, M.D.: The F. A. Davis Co.

ever a capillary tube containing liquid sustains a difference of potential between its extremities, liquid is transferred through the tube towards the cathode. The fact that the flow is proportionate to the difference of potential with a given current is equivalent to the usual statement that the effect increases with the resistance of the septum, because, the greater the resistance, the greater the difference of pressure that a fixed current will establish between the surfaces. . . .

"The following facts explain the character of the process in a clearer and more popular form: If two compartments separated by a membrane are filled with a fluid and in each an electrode is placed, there is a streaming of the fluid through the septum in the direction of the galvanic current,—that is, from the positive to the negative pole,—so that in the course of time there is an increase of fluid in the negative compartment. This osmosis, as is well known, occurs naturally without the use of electricity between two dissimilar liquids, the direction of the osmotic current being from the lighter to the denser liquid. But if the anode is placed in the denser liquid and the cathode in the lighter, this natural osmotic current is not only overcome, but reversed. Du Bois Raymond termed this the cataphoric action of the constant current. This streaming movement is analogous to that taking place in the semi-solid sarcous substance of muscle when subjected to the constant current, and observed under the microscope,—a visible flowing of the contents of the muscular fibre from the positive to the negative pole, causing a swelling of the fibre at the negative end. Now, it has been found that the skin of animals is permeable to drugs. The degree of absorption varies in different animals, and depends upon the quality of drug employed and the manner of its application to the skin. Thus, the skin of the frog absorbs water or watery solutions rapidly, while that of man does not do so at all, because of the fat normally present upon the epidermis and in the pores. Solutions containing alcohol, ether, or chloroform, by removing the fat, render absorption easy. All substances which are volatile and corrode the epidermis, like carbolic acid, are readily absorbed. . . .

"The cataphoric action of electricity has often been made use of experimentally to introduce drugs into the system through the skin. The anode, moistened with a solution of strychnine, has been applied to the skin of a rabbit, the cathode being placed upon any indifferent spot, and in a few minutes the animal has died of strychnine-poisoning. In man, quinine and potassium iodide have been thus introduced and subsequently detected in the urine. . . .

"Efforts have been made more particulary, however, for the purpose of producing a local anæsthesia by the use of electro-chemical osmosis. As far as I can learn, the first investigator in this direction was Dr. B. W. Richardson, who wrote two articles, 'Voltaic Narcotism,' in 1859. He began experimenting in October, 1858, by producing a local anæsthesia with a solution of morphine on the anode. Then, with a simple galvanic current and with tincture of aconite on the positive pole, he brought about complete anæsthesia of a rabbit's ear, after trying in vain to do so with the current alone. After this he made the following solution:

℞ Tinct. aconiti, ℥iii;

Ext. aconiti alc., ℈i;

Chloroform, ℥iii.—M.

"One-third of this was put on a sponge, which was wrapped around the upper part of the hind leg of a dog, after shaving it, and attached to the positive pole, the other pole being applied to the ankle. In eleven minutes there was complete anæsthesia to transfixion of pins anywhere between the electrodes. A minute later a subcutaneous section of the tendo Achillis was made without pain. At the end of an hour the limb was amputated about an inch below the knee without a wince until the bone was sawed, when the animal screamed once, but it was not known whether this was from pain or terror. There was no pain in the subsequent manipulations. Twenty minutes later the animal ate heartily and walked about unconcernedly upon three legs. The wound healed by first intention. Subsequently, transferring his experiments to mankind, he painlessly removed a one-inch nævus from the shoulder of a ten-weeks-old babe, after half an hour's voltaic narcotism, with five minims each of chloroform and tincture of aconite."

Richardson's articles aroused a storm of opposition, the correctness of his views was denied, and Richardson himself abandoned his position. The matter slumbered till 1886, when cocaine anæsthesia by cataphoresis was suggested by Wagner, in Germany, and others followed, so that the literature on the subject soon increased.

Peterson says, "My own early experience with electric cataphoresis extended over many months of time in 1888, and my experiments numbered much over a hundred, about a fourth of which I reported in detail in 1889. Actuated by the great diversity of opinion among the investigators I have quoted, I began at the foundation, in order to demonstrate to my own satisfaction the actual facts in a scientific manner."

Some of Dr. Peterson's experiments I wish to quote, following them with some of my own.

"EXPERIMENT NO. 6.

"*No Anæsthesia with Cocaine Alone.*—This experiment was undertaken for the purpose of ascertaining if the electric current really had any effect in hastening anæsthesia. A four-per-cent. solution of cocaine was applied to the skin of the dorsal surface of my left hand for twelve minutes by holding the open mouth of the bottle containing the solution against it. The skin surface in contact with the solution was one centimetre and a half in diameter. No anæsthesia of any kind was produced.

"EXPERIMENT NO. 7.

"*None with Ten-per-cent. Solution.*—For same purpose as No. 6. Soaked a metal sponge covered electrode, two centimetres square, in a ten-per-cent. solution of cocaine, and applied to skin over first interosseous of my left hand for ten minutes without current. No anæsthesia whatever produced.

"EXPERIMENT NO. 8.

"*No Anæsthesia with Current Alone.*—Same electrode as in No. 7 to same place. A large sponge covered wire-netting cathode, some eight by twelve centimetres square, in the palm of same hand. Sixteen cells of a Grenet battery for six minutes without cocaine. No anæsthesia whatever.

"EXPERIMENT NO. 9.

"*Current and Cocaine together cause Marked Anæsthesia.*—Same electrodes and application as in No. 8, but with ten-per-cent. solution cocaine on the anode. Contact for five minutes with sixteen cells of Grenet battery. Complete anæsthesia to touch, pain, and temperature lasting over an hour in surface in contact with anode. It is known that the anode normally paralyzes the vasomotor nerves, producing congestion and œdema, while cocaine has the opposite effect, contracting the capillaries; but, when applied together, the normal electric effect outbalances that of cocaine, and the part under the anode remains congested. The current itself with this strength is somewhat painful at first at the anode, but as anæsthesia appears, the pain gradually diminishes and ultimately disappears.

"EXPERIMENT NO. 10.

"Exactly same details in every respect as in No. 9, but applied to the hand of Dr. J. A. Booth instead of my own. Same anæsthesia as on myself. Repeated this the next day on Dr. Booth for seven minutes, with same result. The anæsthesia in these short applications is always limited to the area of the anode.

"EXPERIMENT NO. 11.

"*No Effect with Cathode.*—Exactly same electrodes and application as in No. 9. Same number of cells, but commutator reversed so that the ten-per-cent. solution of cocaine was on the cathode. Contact of current for five minutes. Result: continual increase of pain at spot under cathode and no anæsthesia whatever,—the very opposite effects produced by the anode.

"I had now satisfied myself that a watery solution of cocaine alone applied to the skin, or upon the cathode with a strong continuous current, and that the current alone without cocaine, could produce no cutaneous anæsthesia, but that there was an actual cataphoresis of the cocaine solution, and consequent anæsthesia of considerable duration with the anode. I then proceeded to make therapeutic use of this method.

"EXPERIMENT NO. 12.

"*Successful Use of Cocaine; Cataphoresis in a Case of Supra-orbital Neuralgia.*—Dr. E. C. Seguin kindly placed at my disposal an obstinate and severe case of right supra-orbital neuralgia. L. E., female, aged forty years. Duration of neuralgia, a year and a half. Everything tried unavailingly. Suffering every few minutes with the usual agonizing pains of the disease. There was slight analgesia over right half of the forehead and right side of the nose, but exquisite hyperæsthesia of the tactile sense in the same areas, so that a slight touch or breath of air was exceedingly painful. There was great tenderness on pressure. The two-centimetre square anode with a ten-per-cent. solution of cocaine was placed in the right supra-orbital region over a painful spot, and the eight by twelve centimetre sponge and wire cathode in the right palm. Nine Grenet cells were used for three minutes, then raised to eleven cells for two minutes longer, which caused prickling, but no pain; and then raised to thirteen cells, which was too painful, and was reduced to twelve cells, and continued for five minutes. The whole application lasted ten minutes without break of current.

There was no neuralgic pain during this time and the hyperæsthesia had disappeared. There was the expected anæsthesia. The patient was completely relieved from pain for from four to five hours. . . .

"EXPERIMENT NO. 27.

"*Cocaine and Aconitine Cataphoresis.*—I now thought it best to try some further experiments upon myself. A six by ten centimetre cathode was placed in the left palm, and the one centimetre anode over the left first interosseous, as before. The anode was well saturated with a mixture of aconitine and cocaine, as in the last experiment. Twelve chloride of silver cells; five minutes application. Result: complete anæsthesia to touch, pain, and temperature over a space three times the area of the anode, without burning sensation as when aconitine was used alone. There was at first great pallor of skin beneath the anode, which gradually gave way to slight redness in a quarter of an hour. There was slight return of tactile sense in fifteen minutes, and in twenty minutes slight hyperæsthesia to touch, temperature, and pain.

"EXPERIMENT NO. 28.

"*Trial without Current.*—Same electrode, saturated with same solutions as in No. 27. Held it on the back of my left hand without the galvanic current for eight minutes, frequently rubbing in order to hasten absorption. There was no effect whatever, save redness produced by rubbing; no anæsthesia.

"EXPERIMENT NO. 29.

"*Very Mild Current for a Longer Period of Time Successful.*—As in some of the experiments here described unpleasantly strong currents had been used, I tried upon myself the effect of a mild current for a longer period of application than usual. Same electrodes, points of contact, and solutions as in No. 27. Six chloride of silver cells; current imperceptible; ten minutes duration. Marked anæsthesia to pain, touch, and temperature resulted as before.

"When I took up the subject of anodal diffusion, in the winter of 1888 and 1889, the bulk of information at my command was limited and uncertain. There was so much controversy on every point, so much diversity of opinion, that even the existence of the cataphoretic power of electricity seemed to be as yet not scientifically proved. I therefore made some hundred experiments upon myself and patients, the most important of which have already been described above.

"The results of these experiments were summed up as follows: the current alone does not produce anæsthesia at either pole, although the anode has a transitory soothing effect over painful foci. A watery solution of cocaine applied to the skin is not absorbed, does not produce anæsthesia, except, perhaps, after an indefinitely long period. The same is true of chloroform and of an alcoholic solution of aconitine. A watery solution of cocaine is diffused through the skin and subcutaneous tissues by the anode, but not by the cathode. This is true of chloroform, aconitine, strychnine, potassium iodide, corrosive sublimate, tincture of iodine, and other medicaments. The anæsthesia produced by a ten- to twenty-per-cent. solution of cocaine on the anode is sufficient for small operations, and affords distinct relief for from four to eleven hours in cases of severe neuralgias in superficial nerves, and without constitutional effects."

I would recommend to practitioners, having occasion to deal with obstinate neuralgias, a careful study of Dr. Peterson's article.

My own first experiments were not accurately enough recorded to make it worth while to report them; suffice it to say that they demonstrated that the dentine could be affected by the cocaine and the current together. Some later experiments I will record here for comparison with those just quoted.

*No. 1.*—A fifteen-per-cent. aqueous solution of cocaine on cotton wound on positive electrode, applied to back of right hand, at a point where the sensibility to the prick of a fine instrument had been tested, the negative electrode, wet with a salt solution, applied to the palm of same hand. One milli of a seventeen volt current passed for ten minutes. Result: absence of superficial sensation, and I was able to pass instrument through the skin without pain over the area covered by the positive electrode.

*No. 2.*—A fifteen-per-cent. aqueous solution of cocaine applied on cotton to corresponding point and under the same conditions on back of left hand; no current used. At the end of ten minutes there had been no appreciable modification of the sensitiveness.

*No. 3.*—A ten-per-cent. salt solution on cotton on positive electrode applied to another tested point on back of left hand, and negative sponge electrode wet with same solution applied to palm of hand. Current applied for ten minutes, reaching a maximum of thirteen volts and about four millis. There was so much pain and burning as to make this a disagreeable experiment to try. Result: redness and irritation, but no effect upon the sensitiveness.

*No. 4.*—A fifteen-per-cent. aqueous solution of cocaine on cotton was placed in a moderately sensitive cavity of decay in a tooth for fifteen minutes. No modification in sensitiveness took place.

*No. 5.*—Same case; same day. A fifteen-per-cent. aqueous solution of cocaine on cotton, placed in another moderately sensitive cavity for ten minutes, with no resulting modification of sensitiveness.

*No. 6.*—Rubber dam applied, and twenty-five-per-cent. aqueous solution of cocaine applied on cotton to a very sensitive bicuspid cavity of medium size for twenty minutes, without the slightest effect upon the sensitiveness.

*No. 7.*—Same cavity as No. 6. Rubber dam in place. A twenty-five-per-cent. aqueous solution of cocaine on cotton in cavity; positive platinum electrode applied to cotton, and negative sponge electrode to the cheek. Electric current passed for thirteen minutes, beginning with a voltage of three, and attaining a maximum of fourteen and a half, and a maximum quantity of about two-thirds of a milli. Result: absolute freedom from all sensation to the vigorous use of an excavator for removal of decay, and to the use of bur for forming ample retaining grooves in cervical and side walls.

*No. 8.*—Sensitive buccal cavity in inferior third molar. Rubber dam in place. Positive electrode applied in cavity to cotton saturated with twenty-per-cent. sodium chloride solution. Current applied for twelve minutes, reaching a maximum of eight volts and one milli. Much diffused pain resulting while current was applied, but no anæsthetizing effect.

*No. 9.*—Same tooth and same conditions, with the exception of a ten-per-cent. sodium chloride solution. The current applied for ten minutes, reaching a maximum of nine volts and one and one-half millis. A severe pain extending around to the opposite bicuspid region and into the neck during application. No appreciable lessening of sensitiveness.

*No. 10.*—Same cavity. A fifteen-per-cent. aqueous solution of cocaine instead of the salt solution. Current applied during eleven minutes, reaching a maximum of ten volts and about two-thirds of a milli. No diffuse pain, a very little in tooth. Result: a marked lessening of sensitiveness of whole cavity, and entire removal of it in a large part of cavity.

As a rule, this class of cavity requires longer applications than approximal cavities, and it is sometimes difficult to affect that part which is out of the path of the current.

*No. 11.*—Coronal cavity in left superior second molar. Quite sensitive. Placed in a half-per-cent. solution of sodium chloride on cotton in the cavity and applied current for ten minutes, beginning with a voltage of less than one, increasing it to eight and a half at the end of the ten minutes. The milliampère metre then recorded about a half milli. I was unable to work the current up faster because of the pain it caused. The sudden turning off of the current at the end of the ten minutes gave a very decided sensation (kick or kink, patients often call it) in the tooth. The sensitiveness of the dentine to the excavator had not been in the least diminished.

*No. 12.*—Same cavity as No. 11. A twenty-five-per-cent. aqueous solution of cocaine applied in this way, beginning with same voltage. After the first two or three minutes it was possible to increase the voltage much more rapidly, without undue pain, than was possible with the salt solution, this fact being seemingly due to the beginning anæsthetic effect of the cocaine. At the end of eight minutes seventeen volts (or twice the quantity given in No. 11) was being administered and with less pain. At the end of ten minutes there was no pain at all. The milliampère metre recorded about three-fourths of a milli. When the current was suddenly cut off, there was much less of the "kink" noted by the patient,—another indication of deep cocaine effect. Upon vigorous use of the excavator, using the same instrument as before and on the same place, the dentine was found *absolutely* free from all sensation. The patient, a girl of about fifteen, in response to close questioning, assured me that there was no sensitiveness whatever to the thorough excavation of the cavity after the use of the cocaine, whereas she flinched very promptly under careful efforts at removal of the decay with the same instrument before any application of cocaine had been made, and also after the application of the electric current alone, using the salt solution only for the purpose of making good electrical connection. It should be said that this patient and the one for Experiments Nos. 6 and 7 had never had the process used before, and that no explanations were made beyond the statement that I was going to try to relieve the sensitiveness, and that they should not be hurt.

The results of these experiments may be summed up as follows: Ten-per-cent. aqueous solution of cocaine applied on the positive electrode with a weak electric current for a few minutes will anæsthetize the skin. (Experiment No. 1.)

A similar cocaine solution applied for the same time without the current has no anæsthetic effect. (Experiment No. 2.)

The same electric current without the cocaine has no anæsthetic effect. (Experiment No. 3.)

Cocaine solutions of from fifteen to twenty-five per cent. applied in sensitive cavities, for ten or twenty minutes, do not modify their sensitiveness. (Experiments Nos. 4, 5, 6.)

The electric current alone applied to sensitive dentine, sodium-chloride solutions of varying strengths being added to insure good electrical connections, does not perceptibly modify the sensitiveness. (Experiments Nos. 8, 9, 11.)

Cocaine solutions and the electric current applied to sensitive dentine, together, *do* completely anæsthetize it; consequently the cocaine is the active agent. (Experiments Nos. 7, 10, 12.)

It seems probable that the exhibition of other drugs may produce the same results, and I have other experiments preparing to demonstrate the facts concerning certain other drugs. These I hope to find worth reporting later. I hesitate less about reporting results with cocaine without lengthy trial than with most other drugs, since, theoretically, it can do no possible harm to the tooth.

The effect of the cocaine in these applications does not seem to reach deeply into the dentine in most cases. By prolonging the application, however, the pulp itself may, in favorable cases, be anæsthetized, even through a layer of dentine.

It is quite often the case that a ten- or twelve-minute application will anæsthetize the dentine deeply enough to allow the greater portion or all of the cutting to be done painlessly, but for deep grooves it may be necessary to repeat the application. In cases where there is much sensitiveness, and consequently much time will be required to prepare the cavity at all, I find the time required for applying the cocaine is fully made up by the increased speed possible after the sensitiveness is under control.

I do not need to enlarge upon so familiar a fact as the comparative consumption of time in preparing cavities, similar, except for their degree of sensitiveness, or to recall how, in the case of a sensitive cavity for a nervous patient, it often requires an hour to do what may be done in ten minutes after the cavity has been thoroughly anæsthetized.

This treatment renders it possible to do for these nervous and hypersensitive organizations desirable operations, which, without some such means, are utterly impossible. It also enables the operator to perform much more satisfactory operations for sensitive children from twelve to sixteen years of age, and to do these operations (which would otherwise be entirely too formidable to contemplate) with no objection from the little patient and with no danger of exhaustion afterwards.

Holding, as I do, very decided views in favor of thoroughly performed operations, I find much relief from being able to perform such operations without regard to sensitiveness.

Of course, the operator who knows no difference in cavities, but selects a bur at the beginning, and completes the preparation of his cavity in one fell swoop, will not have much use for an obtunding process which requires time to apply. But for the careful operator, careful alike of his patients' best interests, his own reputation, and the honor of his profession,—for him I am confident that this process is to become a valued one.

As to the time the cocaine effect persists, I find it difficult to get accurate knowledge; I am not always able to be certain whether renewed sensitiveness is due to penetration through the anæsthetized layer or to returning sensation. I had expected that the effect would be more lasting than cocaine effects in tissues, where the circulation is more rapid. I have, however, seen one or two cases where there had been a profound effect produced and where there was a return of the sensitiveness in fifteen or twenty minutes.

As to the effect upon the pulp or the tooth, I have examined some of the teeth where the first applications were made, and I am unable to find any trace whatever of a permanent change in their condition.

With regard to the effect of the current itself, Dr. J. S. Marshall's[1] article in the American Academy's *Transactions*, and his continued use of applications of electricity for morbid and irritable conditions of the pulp, will perhaps be reassuring to those who fear ill effects upon the pulp.

Some of my first work with cocaine and electrical osmosis was done in my own mouth; stronger currents were used than I dare to apply in the mouths of my patients, and deep anæsthetic effects were obtained. No ill results have followed, and the tooth is normal in its sensitiveness after nearly a year's test. As to the applicability of the method, I find that it is almost universal. The exceptions are in the cases of the comparatively rare subjects who are not readily affected by cocaine, and the occasional case where the difficulties of insulating the tooth or cavity are too great.

This latter class of cases will be much smaller in the hands of the expert operator, and this added necessity for the rubber dam

---

[1] Electricity as a Therapeutic Agent in the Treatment of Hyperæmia and Congestion of Pulp and the Peridental Membrane.—*Dental Cosmos*, November, 1891.

will stimulate the operator's ingenuity in applying it in difficult cases. I very seldom find a case where I am unable to use the method with success, if I desire to do so.

In my use of electrical osmosis I have found a single patient who is so sensitive to the current as to be only able to take a seven volt current as a maximum. For her I readily anæsthetized a bicuspid so as to remove a portion of the pulp, but a large molar cavity required a long application, and was only a partial success.

As to deep anæsthetic effects about the roots of teeth, I have not met with success in the limited trials I have made. I have received numerous queries as to the use of the method for extracting, but I see no probability of its replacing the present methods for that purpose. I have made one or two attempts to reach the nerve-branches entering the tooth by applying the electrodes on opposite sides of the gums. The results were not encouraging, and in the case where my most determined efforts were made, I produced a decidedly objectionable result in the breaking down of the gum tissue under the positive electrode. I have, however, obtained satisfactory results from cocaine cataphoresis on the sensitive gum about roots undergoing preparation for crowning.

It would seem to me feasible to treat by electrical osmosis that very troublesome condition resulting from undue wear or erosion of the grinding surfaces of molar teeth, where the sensitiveness is almost a menace to health, by reason of its preventing proper mastication, and where it defies the action of the most violent caustics.

For doing this work I have had made the instrument you see before you, technically an adapter or fractional volt selecter, the working of which I will explain.

A milliampère metre is also almost essential. This selecter is intended for use with the Edison one-hundred-and-ten-volt continous current. I have the Electro-Therapeutic Company at work devising a battery apparatus for use instead of the Edison current. I have not used batteries because I did not want to have the care of them, but when the incandescent current is not accessible, battery systems with a capacity of thirty or more volts may be successfully used with suitable modifying apparatus.

First, let me enumerate the precautions necessary in using the one-hundred-and-ten-volt current.

Have the selecter connected according to the instructions provided with it.

An absolute safeguard, but one not necessary if connections are properly made, is to insulate the chair by placing linoleum or rubber under its feet, and also to see that gas-pipes, water-pipes, and any other wires are out of reach or protected from contact.

I always apply the rubber dam, as it is difficult, and often impossible, to prevent leakage of current through other tissues if this is not done.

Any metal fillings which will be in contact with the wet cotton in the cavity or with the electrode must be covered. The current from a metallic surface into dentine is irritating and painful. I find Gilbert's temporary stopping a very useful material for this purpose. Wax will also do. In cases where I am working on an approximal cavity in one tooth, and a filling in the next tooth is too close to allow of its satisfactory insulation, I apply the rubber at first only over the tooth to be worked upon, thus insulating it completely; or, if the rubber is already in place a second rubber may be applied over the tooth to be worked upon. The positive electrode should be of platinum, as most other metals are affected by the current, and are liable to stain the tooth.

These conditions being provided, see that your current is turned on. I always test this by touching the metal parts of the electrodes together, and watch the milliampère metre to observe the result.

This selecter is so arranged that when the needle is at zero, and contact of eloctrodes is made as described, about one milli will be recorded. Getting this result assures the operator that all connections have been made, and that the apparatus is ready. If, however, a larger quantity of current is indicated by the milliampère metre, it shows that the rheostat contact is not at the right place. This same proceeding would also serve to detect any breakdown in the rheostat if it had occurred. Twenty seconds serve to assure the operator on these points, if his apparatus is conveniently placed. I then wet the negative sponge electrode with water or dilute salt solution. I place in the cavity a pellet of absorbent cotton saturated with a twenty- to thirty-per-cent. cocaine solution. I prefer not to have this cotton extend outside of the cavity, and to keep the solution confined to the cavity as much as possible. This concentrates the current in the part I desire to affect.

The negative wet sponge electrode I usually allow the patient to hold most of the time. It is preferably to be applied about the face or neck, as near the tooth as is convenient. Having placed this and allowed the patient to take it, I apply the positive electrode to the cotton in the cavity, and begin slowly to increase the current by turning the large fibre knob of the rheostat head in the direction indicated by the needle which records voltage. The first consciousness of the current sometimes comes to the patient as the typical little "kick" or "kink" of the galvanic current, but it is a very small one with this selecter. More often the patient is only conscious of an indefinite, gradually increasing pressure, and if the current is pushed too rapidly this may increase to pain. It is therefore necessary to watch the patient carefully, and to pause in the turning-on process as soon as the change in the eye of the patient indicates that he is beginning to feel the current to an uncomfortable degree.

After the first experience, if cautiously managed, a patient will usually give the operator all necessary indications for his guidance, and allow him to keep the current up to a point just short of pain.

After one experience with it, the sensation is readily born, even by sensitive children of twelve or fourteen. In fact, they are often the most enthusiastic about its use.

As the operator pauses at the point where the patient indicates that he is getting enough, or even turns back a little if there is too much current, it is well to assure the patient that any disagreeable sensation will subside promptly. It usually does this in from one half minute to two minutes, and then the voltage may be increased slowly and gradually, with pauses long enough for any disagreeable sensation to disappear.

Subjects differ very much in the amount of current they will bear without discomfort. It is usually found, however, that by very gradual increase, and by taking more time to reach the maximum in these sensitive cases, a sufficient amount may be applied, to any case, to attain the result of anæsthetizing the dentine.

It is my customary habit, as soon as I have opened into a sensitive cavity, to make an application lasting from eight to twelve minutes. If I have reason to expect difficulty with the case, I make the application longer. If the first application is not sufficient for all I wish to do, I repeat it later.

I have some ten or twelve cases on record where twenty to thirty minutes have been needed to get sufficient effect. These were all cases where both patient and operator felt compensated for the time spent. Most of them were either extremely sensitive teeth or subjects who could bear but little current, and several of these cases would have been all but impossible without the aid of this method.

On the other hand, I have numerous cases where ten or even eight minutes have been ample time for successful results.

Having reached a voltage likely to be sufficient, I allow it to stand at that point till the end of the application.

Fifteen to twenty volts will usually be attained in seven or eight minutes. In many cases, with small cavities and little sensitiveness to the current, twenty-five or thirty volts may be marked in the same time.

The higher voltage works more rapidly.

At the end of the application, I usually break connection at the negative electrode, as there is less often any shock in so doing. If the subject is very sensitive to the current, I turn the voltage down low before breaking connection.

Having concluded the application, I turn off the current in the selecter by means of the switch. This lever may also be used for concluding the application of current if you find no objectionable shock resulting.

Then I test the cavity, and finding it all right proceed as usual, bearing in mind that the effect may not have gone as deeply as I wish to go with my instruments, so it is still necessary to watch for signs of returning sensitiveness.

The expert electrical knowledge required for this process is not such as to be a formidable obstacle to any skilful practitioner. The instructions provided will serve to arm him with sufficient knowledge for his first cases, and the other needed knowledge will come to him quickly as he goes on with his work.

In connecting this selecter, it is only necessary to screw the plug provided into a lamp socket, and place the cords in the binding posts. Carry the wire from the positive binding post to the binding post of the milliampère metre, which is marked + (positive). Connect the cord attached to the positive platinum electrode to the other post of the milliampère metre. Now lead the cord of the negative electrode from the unused post of the selecter.

The electrodes may be readily detected by passing a current through a small piece of wet litmus paper. The positive pole will be found to redden the litmus, while the negative turns it blue.

Case A.—Mr. C. M., aged twenty, reports his teeth as very sensitive, and all cavities, even the smallest, prove so. Inferior second molar buccal cavity. Applied a twenty-per-cent. cocaine solution with electric current for twelve minutes,—seventeen volt current for the final third of the period. Result: entire absence of sensitiveness in the preparation of the cavity. For same patient at same sitting, right superior first bicuspid corona distal cavity, twenty-per-cent. cocaine current for ten minutes, reaching a maximum of seventeen volts. Result: complete absence of sensitiveness except for final groove cutting. As the patient was anxious to be able to say that it had not hurt him at all, a second application was made about like the first, and the cavity completely anæsthetized. One of the most competent New York practitioners, in whose hands the patient had previously been, assured me afterwards that he had used every known remedy without success in the effort to even relieve the sensitiveness in this case.

Case B.—C. Y., aged fifteen. Mesial cavity in right central incisor, very sensitive. Fifteen-per-cent. cocaine current for eleven minutes up to a maximum of twenty-five volts. Result: complete anæsthesia of the dentine during the preparation of the cavity. Same patient and same sitting. Similar cavity in left central; same treatment, and similar results. The patient went to sleep during this second application, which, when we consider that this was his first experience, is good evidence that the process is not a painful one.

Case C.—Miss E. G., aged fifteen. Approximal cavities between the right superior bicuspids, so sensitive that the patient could scarcely bear the least touch in them. Twenty-per-cent. cocaine and current applied. Dr. C. A. Woodward called after I had begun my application, and I asked him to come to the chair.

I quote this case partly that he may tell you what he saw. In order to make sure of this case, I prolonged the application to twenty minutes. The voltage was not recorded, but it was seventeen or less. I then took a bur in the engine and cut the cavities out thoroughly without the slightest sensitiveness being found.

This is one of the cases which gave me evidence of the time in which sensation may return.

I left the chair for a few minutes, between five and ten, to speak with Dr. Woodward, and when I returned the territory which had not been sensitive when I stopped work was again as sensitive as it was at first.

A second application of eight minutes was made and the shaping of the cavities completed painlessly.

Case E.—Miss M. M., aged twenty-two. Inferior left cuspid, labial cavity, near gum, quite sensitive. Twenty-five-per-cent. cocaine solution, current for seven minutes up to a maximum of thirteen volts. Result: complete anæsthesia of the cavity during its preparation with a bur.

The apparatus, as I use it, is supplied by the Electro-Therapeutic Company, 32 East Twenty-third Street, New York.

I wish to acknowledge the intelligent assistance of their electrician, Mr. G. M. Wheeler, in working out the difficult mechanical details after I had elaborated the principles required for the process and apparatus.

